# Older patients’ autonomy when cared for at emergency departments

**DOI:** 10.1177/09697330221105637

**Published:** 2022-06-21

**Authors:** Catharina Frank, Mats Holmberg, Elin Ekestubbe Jernby, Annika Sevandersson Hansen, Anders Bremer

**Affiliations:** Faculty of Health and Life Sciences, 6140 Linnaeus University, Växjö, Sweden; Centre of Interprofessional Collaboration within Emergency care (CICE), Linnaeus University, Växjö, Sweden; Faculty of Health and Life Sciences, 6140 Linnaeus University, Växjö, Sweden; Centre of Interprofessional Collaboration within Emergency care (CICE), Linnaeus University, Växjö, Sweden; Centre for Clinical Research Sörmland, 8097 Uppsala University, Eskilstuna, Sweden; Department of Ambulance Service, Region Sörmland, Katrineholm, Sweden; Emergency Department, Region Blekinge, Karlskrona, Sweden; Emergency Department, Region Kronoberg, Växjö, Sweden; Faculty of Health and Life Sciences, 6140 Linnaeus University, Växjö, Sweden; Centre of Interprofessional Collaboration within Emergency care (CICE), Linnaeus University, Växjö, Sweden

**Keywords:** Aged, autonomy, emergency department, ethics, nurses, phenomenology

## Abstract

**Background:**

Older patients in emergency care often have complex needs and may have limited ability to make their voices heard. Hence, there are ethical challenges for healthcare professionals in establishing a trustful relationship to determine the patient’s preferences and then decide and act based on these preferences. With this comes further challenges regarding how the patient’s autonomy can be protected and promoted.

**Aim:**

To describe nurses’ experiences of dealing with older patients’ autonomy when cared for in emergency departments (EDs).

**Research design:**

This study adopted reflective lifeworld theory and a phenomenological design.

**Participants and research context:**

A total of 13 open-ended interviews were performed with nurses working at two EDs in Sweden.

**Ethical considerations:**

The study was reviewed by the Ethical Advisory Board in South East Sweden and conducted according to the Declaration of Helsinki. All participants gave consent.

**Findings:**

Nurses’ experiences of dealing with older patients’ autonomy in EDs are characterized by moving in a conflicting uphill struggle, indicating obscure thoughts on how patient autonomy can be protected in an ethically challenging context. The phenomenon is further described with its meaning constituents: ‘Being hampered by prioritization under stress’, ‘Balancing paternalism and patient autonomy’, ‘Making decisions without consent in the patient’s best interests’ and ‘Being trapped by notions of legitimate care needs’.

**Conclusion:**

Stressful work conditions and lacking organizational strategies in EDs contribute to nurses maintaining unjustified paternalistic care, regardless of the patient’s ability and medical condition, and questioning who has legitimacy for participating in decisions about care. The nurses’ protection and promotion of older patients’ autonomy is dependent on the opportunity, ability and willingness to create a patient relationship where the patient’s voice and preferences are valued as important. Consequently, strategies are needed to improve patient autonomy in EDs based on the idea of ‘relational autonomy’.

## Introduction

Older people seeking emergency care often have complex needs and limited ability to make their voices heard. In emergency departments (EDs), this can lead to physical, mental and existential care needs being overlooked, but also that ethical values are not respected. There is an obvious risk that the healthcare professionals does not take into account the patient’s preferences, which constitutes a threat to patient autonomy. As there is a lack of knowledge about older patients’ autonomy in EDs, both from a patient perspective and from a caregiver perspective, this study focuses on how ED nurses deal with older patients’ autonomy.

## Background

Autonomy is considered to be an important value in the western world, usually to in maximizing the ultimate value(s) of autonomy but also as a value per se. Autonomy consists of four components: preference, decision, action and the intermediate relation of ‘because’. In short, this means that, ‘in order for a person to be self-determined, the preferences need to be the person’s own, and the person needs to make a decision to realise or satisfy these preferences and then act on this decision’ (p. 118). The first three components have to be explicitly connected to each other so as to avoid any reduction of autonomy. The fourth ‘because’ component signals the degree to which the first three components are controlled by the person whose autonomy is concerned. In the healthcare context, the patient’s autonomy is central.^1^ Older patients are often considered to be more vulnerable than others, and hence in need of extra attention and protection. On the one hand, this vulnerability is linked to helplessness, neediness and victimhood and is the product of paternalistic and coercive forms of interventions.^2^ On the other hand, vulnerability is an expression of dependence on one another, which must be managed responsibly by the person whose task it is to help the person who is vulnerable,^3^ for example, as a result of an older patient’s acute illness.

The number of older people with multimorbidity who seek emergency care is increasing worldwide.^4,5^ Each year more than 30% of patients aged 80 years or older require care in EDs.^6^ Older patients stay longer in the ED, are more likely to be admitted as inpatients and have higher risk of mortality than other age groups.^7^ They attend the ED with more serious medical conditions now than they did previously. Their visits are related to vague, intermittent and non-specific conditions as well as specific diagnoses (ICD-10), such as pneumonia, malaise and fatigue, heart failure and atrial fibrillation.^6^ The older patients’ care-seeking behaviour is influenced by their condition, its severity and the onset of symptoms, together with patients’ previous experiences of similar symptoms and their significance. Limited access to prompt primary care and social and financial resources has also been shown to influence care-seeking behaviour. Older patients’ decisions to seek ED care are thus complex, influenced by multiple internal and external influences, including their medical condition, together with inappropriate organizational structures and limited collaboration between primary and emergency care. Additionally, the ED environment has been described as being unsuitable for older patients and negatively influences the ability of healthcare professionals to care and address their complex and unique needs,^7^ especially for those affected by severe medical conditions.^8^

In EDs, older patients’ care needs are de-prioritized as a result of other patients being judged to have more urgent/acute medical needs, along with a focus on physical ailments that can be remedied with medical treatment.^5^ As a result, the staff pose short questions to the patient, and expect short answers in return, to exclude the most serious and life-threatening conditions.^9^ After an initial assessment, older patients often end up being alone and without regular contact with staff. This generates feelings of being invisible, abandoned and unconfirmed.^10^ Older patients describe a lack of being kept informed, which creates uncertainty about whether the condition is serious, what they can manage themselves, who is responsible and to whom they can ask questions.^11^

Older patients may be affected by impaired cognitive ability, and decision-making can be bewildering and stressful and may lead to the patient becoming agitated when uncertain and important decisions have to me made under time constraints.^12^ Staff may also feel uncertain about how they can meet the existential needs of older patients.^13^ The choices are often made by the staff due to lack of time and without speaking with the patient, which makes the patient feel unable to influence what is happening,^9,14^ even when staff try to adapt their communication approach and involve the patient’s family.^9^ The asymmetric distribution of power within a staff-patient relationship prioritizes the professionals. While a shift in the distribution of power from staff to patient is desirable, there is a risk that nurses and other professionals may develop misconceptions about situating autonomy in relation to the patients’ abilities to always be active, self-managing and capable of making the right choices for themselves.^15^ Instead, it is argued that patient autonomy should be viewed as being positioned within a responsible relationship with others where professionals’ intervention in the decision-making process is understood as a prerequisite for autonomy.^16^ Such ‘relational autonomy’ is thus based on a commitment from professionals to protect and promote the patient’s autonomy in making judgements that are true to the patient’s own wishes and values instead of abandoning the patient to make such choices themselves.^3^

Lack of participation for older patients has been described as an ethical issue that mainly concerns physicians,^17^ and often staff fail to allow patients to participate in the care process.^18^ Staff may act consciously but are reluctant to accept the patient’s wishes and preferences,^18,19^ believing that they do so in the patient’s best interests. At the same time, patients often feel powerless and dissatisfied with ED care,^11^ perceiving that they are not invited to participate in their own care, which contributes to a lack of autonomy and deprivation of self-determination.^20^ Shortcomings in ED care have been attributed to non-compliance with the principle of autonomy, and patient autonomy is limited, or lacking, when nurses’ own values and attitudes solely govern the care they provide.^18,20^

Given that older patients often have complex needs and may have limited ability to make their voices heard, there are ethical challenges for staff to establish a trustful relationship to find out the patient’s preferences and then decide and act based on these preferences.^20^ Therefore, ED nurses need insight into varying abilities in older patients to be autonomous, especially in patients affected by dementia or confusion, where the ability to make choices can fluctuate.^14,21^ However, limited numbers of studies explore how ED nurses experience older patients’ autonomy, a knowledge base that should be expanded to meet the rights and needs of a growing population of older persons worldwide. Thus, the aim of this study was to describe nurses’ experiences of dealing with older patients’ autonomy when cared for in EDs.

## Research design

This qualitative study adopted a reflective lifeworld theory and phenomenological design.^22^

### Participants and research context

This study was conducted in two EDs in south Sweden. One region is 8466 square kilometres in size, with 201,469 inhabitants (24 inhabitants/sq km). The other is 3039 square kilometres in size, with 159,606 inhabitants (53 inhabitants/sq km).^23^ In total, approximately 67,000 adult patients were cared for at the two EDs in 2018.

Using purposive sampling,^24^ the ambition was to recruit a sample that varied by gender, age, experience and education among Swedish-speaking registered nurses (henceforth referred to as ‘nurses’). The recruitment was conducted by the head nurses at the EDs, who informed and asked nurses about voluntarily participating in the study. Inclusion criteria were nurses (general and specialist), who had at least one year’s experience working at an ED. Information about the study was distributed by email to each nurse, and verbally provided by the head nurses. Interested nurses were encouraged to establish direct contact with researchers responsible for data collection and interviews (the third and fourth author). In total, 13 nurses agreed to participate. All participants were women (mean age 42 years, median 42 years). No male nurses agreed to participate. Their length of experience of working as a nurse in EDs varied from one to 38 years (mean 15 years, median 11 years), see Table 1.

**Table 1.** Demographics of study participants (*n* = 13).

	Gender	Age (years)	RN^a^ specialist education	Experience as RN (years)	Experience as RN in ED^b^ (years)	Care experience before RN	Geriatrics education^c^
1	♀	62	None	38	31	Nursing asst.	Yes
2	♀	49	None	27	25	Nursing asst.	No
3	♀	51	Emergency	15	10	Nursing asst.	No
4	♀	34	Emergency	11	12	Nursing asst.	No
5	♀	27	None	2	2	Nursing asst.	Yes
6	♀	26	None	1	1	None	Yes
7	♀	59	None	25	11	Nursing asst.	Yes
8	♀	31	Emergency	8	1	Care asst.	No
9	♀	29	None	7	2	None	No
10	♀	48	None	28	20	Nursing asst.	Yes
11	♀	58	None	22	20	Nursing asst.	No
12	♀	28	None	5	3	Nursing asst.	No
13	♀	40	Emergency	7	7	Nursing asst.	Yes

^a^ RN = registered nurse.

^b^ ED = emergency department.

^c^ Including experience from geriatric ward.

### Data collection

The data collection was carried out with open-ended individual interviews to stimulate openness to the phenomenon, that is, older patients’ autonomy in the ED.^22^ All interviews were conducted in January 2019. The interview questions were developed and tested in three pilot interviews with nurses. The pilot interviews provided the interviewers with experience in conducting the interviews, and the questions did not need to be adjusted. The pilot interviews were not included in the results. Each interview was introduced with the intention of promoting a dialogue about autonomy in older patients. Before posing the questions, the participants were informed that the topic of the interviews concerned older patients. The interviews were introduced with two opening questions: ‘Can you talk about a situation when you involved the patient?’ and ‘Can you talk about a situation when you made a decision for the patient?’ By asking these two open-ended questions, the intention was to capture the nurses’ experiences of dealing with older patients’ autonomy, between one question aimed at patient participation/self-determination and the other at paternalism/patient’s best interests. Follow-up questions were then used to further deepen the discussion, such as: ‘How do you mean?’, ‘In what way?’ and ‘Can you elaborate on that?’ All interviews were conducted in a separate room away from the ED, at a time and place suitable to the participants. Each interview lasted between 12 and 32 min (median 26 min) and was digitally audio-recorded.

### Data analysis

The interviews were analysed based on lifeworld theory with a descriptive phenomenological approach.^22^ The purpose with the data analysis was to describe the essential meaning of the phenomenon, based on the ED nurses’ lived experiences, in this study, of dealing with older patients’ autonomy in the ED. The analysis process followed a flexible non-linear movement between the whole and the parts. Initially, the interviews were respectively listened to by the two interviewers several times, followed by repeatedly reading the transcripts, to become familiar with the data as a whole. This initial part of the analysis was followed by a first screening of the data with a focus on finding sentences and paragraphs responding to the aim. Meaning-units were extracted, sorted and compiled into preliminary clusters of meaning (*n* = 31). The initial number of clusters was then compared in relation to differences and similarities and, finally, compiled into seven clusters of meaning. The meaning of each cluster was then further examined to describe the essence as a starting point to form a new whole.^19^ The first, second, third and fourth author took part in this phase and, while writing about the essence, variations emerged in the meanings, which were brought together in meaning constituents. The analysis process was based on remaining open to the phenomenon and by critically reviewing one’s own pre-understanding, so that a new whole could be discovered and described. This critical reflection of the authors’ pre-understanding was achieved in a dialogue between all authors and in seminars with other peers. The essence of the general structure of the phenomenon is found in its description, together with the description of the meaning constituents, as supported with quotations from the data.

### Ethical considerations

The study was reviewed by the Ethical Advisory Board in South East Sweden (EPK 540-2019). Throughout the research process, the principles of the Declaration of Helsinki^25^ were considered and applied. All participants were informed that their participation was voluntary and that they could withdraw at any time without providing a reason, and each signed a written consent form prior to the data collection.

## Findings

**Figure 1.**
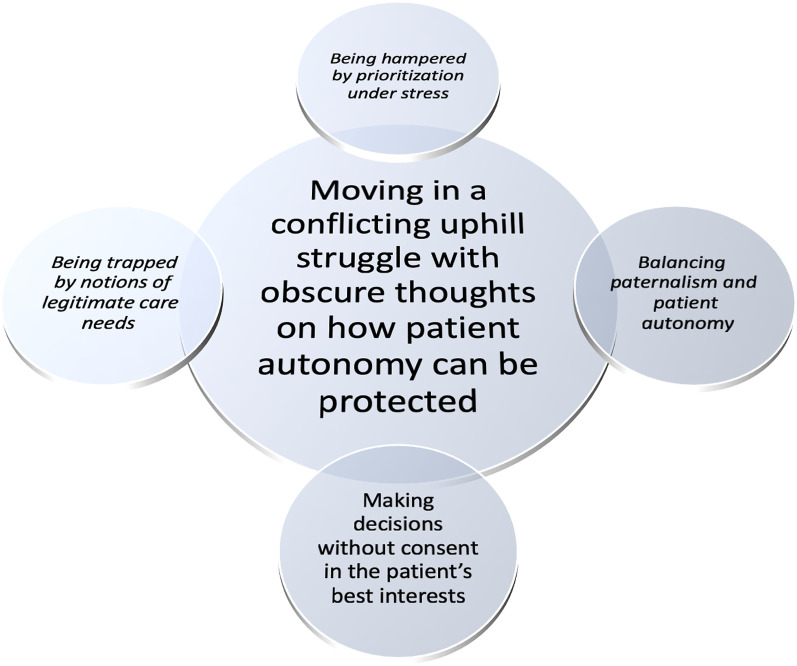
The essence and general structure of the phenomenon.

Emergency department nurses’ experiences of dealing with the older patients’ autonomy means a conflicting uphill struggle indicating obscure thoughts on how to protect the patients’ autonomy in an ethically challenging context (Figure 1). The starting point was their own values, based on providing emergency medical care only, which presented a risk that responsibility for promoting the patients’ autonomy became unrecognized, unnoticed or not understood. The older patients’ autonomy was thus handled in an uncritical and non-reflective way, although with an ambition to act in the patient’s best interests. The preservation of the older patients’ autonomy was hindered by a lack of ability to critically reflect on ethical problems and limited access to patients’ informed consent. A lack of understanding of how to support and promote patient participation in an aggravated environment violated this fragile autonomy, leading to inadequacy in the professional role. The coercion implied in disregarding the patient’s autonomy in emergency situations risks being applied in all care relationships, regardless of the degree of urgency in the patient’s condition.

The general structure of the phenomenon is further described with the meaning constituents; *‘Being hampered by prioritization under stress’, ‘Balancing paternalism and patient autonomy’, ‘Making decisions without consent in the patient’s best interests’* and *‘Being trapped by notions of legitimate care needs’.*

### Being hampered by prioritization under stress

In a care environment that was calm and without time-pressure, critical reflection about promoting the patient’s autonomy was supported and the older patient’s autonomy was respected. It was an endeavour to establish a relationship characterized by openness and compliance with the older patient’s abilities and, hence, let the patient make their own decisions, when this was considered possible or appropriate. The patient’s confirmation about planned nursing care activities was sought through support, helping and testing their abilities. In this there was a need to be prepared to interrupt nursing care activities if the older patient opposed, by being sensitive in the conversation and observation of the patient.

The care environment was often aggravating and stressful due to lack of time and resources. Thus, there was a lack of critical reflection about how to involve the older patient in the care and promote participation whenever possible. Together, these created a breeding ground for despair for the nurses in involving the patient, thereby threatening patient autonomy.

We barely have time to say hello to the patient and then it is quite difficult to get him/her involved in what happens and happens. So it is probably a lack of time that means that you do not have that time in the emergency room to sit down and talk to the patients. You should be happy if you have seen your patients on a shift, unfortunately. It affects the patient in such a way that they do not get … The nursing care is not as good as you want it to be, because it is neglected because it is medical things that you need to do that are more important.

When the nurses were exhausted or experienced the older patient as demanding, the patient was not involved in the nursing care. Ethical reflection was absent and patient autonomy threatened. Prioritizing under stress meant that nurses disregarded their responsibility to protect the patient’s autonomy or were not able to take responsibility that was perceived as laborious and extensive. The nurses were hampered by the stress and felt compelled to take an approach to the patient in which the patient’s autonomy was de-prioritized in the patient-nurse encounter, leading to the exclusion of patients and deficiencies in nursing care.

### Balancing paternalism and patient autonomy

When the atmosphere was open and permissive, with time and opportunities for calm conversations, explanations were given to allow the patient to understand the limitations and risks of the choices considered. However, these conversations were based primarily on demands to direct the patient towards predetermined emergency medical goals, without any, or with limited, ambition to sensitively listen to the patient’s will.

In the triage, it is very much from the patient’s horizon; that they come here and they describe their problems. And it will be a lot on their terms, but that you try to explain and lead them, that is, that you lead them to different things, partly in the triage when you think that… When they seek care here, because then it is always this that one tries to meet their questions and arguments if I may put it that way and try to explain what the width is. And then it really becomes that we are the ones who lead them to certain things.

When explanations, advice and information turned into persuasion, the atmosphere in the encounter became closed, controlled and only mildly compelled to obtain patient approval. With this came attempts to one-sidedly argue for one’s own perspectives and motives, meaning that the patient’s preferences were not respected, with the consequence of creating mixed feelings of having both done the best for the patient and disregarded the patient’s will.

In the ED environment, unknown to the patient, a preventive approach was taken towards the patient’s autonomy. This meant making choices and decisions based on the nurses’ own values, albeit in the best interests of the patient, to protect the patient and prevent harm and injury. The nursing care was guided by the nurses’ own understandings of what they themselves, as patients, or their significant others, would expect as good care in similar situations. However, there was still uncertainty about the patient’s authentic will.

Usually you try to do certain things for their own good. But you still do not know what their best is always. It is well-meaning, as if you might, I want to help them. But you take it for granted that all people want things, but I can still not know.

Nurses’ own ethical values and experiences were also used as a foundation to protect patient autonomy by questioning decisions made by other professionals (e.g. physicians and nurse colleagues), requiring nurses’ courage to protect the patient from harm or added suffering. In such situations, the nurses assumed that the patient did not understand what was best and their autonomy was suspended with the intention of protecting them. This meant that patient autonomy was limited because of the influence of the nurses’ own ethical values. However, the inclusion of significant others was experienced as strengthening patient autonomy in some situations, thus providing important information about the patient’s wishes.

### Making decisions without consent in the patient’s best interests

In care situations when nurses showed inability for ethical reflection and ignorance, the patient was not given space or opportunity to participate. In such cases, where the treatment of the patient’s disease was urgent, or where impaired cognitive ability contributed to unexpressed wishes and opinions, the nurse made decisions without gaining permission from the patient. Further, it was perceived that the patient implicitly consented to transfer such decisions to the nurses, in being judged as not able to make decisions independently or participate in their own care.

They [the patients] are used to obeying and in that situation you are some kind of authority. (…) I usually want to think about my decisions, but it is not often that you give patients that time.

In addition, any decisions made in the ED were temporary in urgent situations where patients could not be independently involved. Patients who were unconscious or had limited decision-making ability had no opportunity to express authentic wishes and the nursing care was based on what was judged as being best for the patient, based on medical data and the information available about the disease state, and the patient’s previous status.

Very old people can be very affected by fever or something else and then it is we who control. We do not say ‘do so, so, so’ … but you explain and you tell. You take off the clothes and it is… The patient does not really have much to decide about there, I experience, but it is we who control the process. Yes, so it’s not appropriate either. I’m just trying to think like, at all… No, it’s us who decide. The patient does not really have much to say about it.

Decisions were made without consent, often to provide lifesaving interventions, forcing the nurses to act based on what was believed to be in the patient’s best interests. Here, the patient was not considered as having autonomy, and the nurse was forced to temporarily take on a paternalistic role.

### Being trapped by notions of legitimate care needs

The approach to the older patient’s autonomy was also threatened by notions about the care assignment and which care needs and patients are to be considered legitimate in ED. When the patient was judged to be in an inappropriate level of care (e.g. requiring primary care instead of emergency care), the nurses led, influenced and controlled the decisions in a direction that did not necessarily benefit the patient but instead the nurse’s work situation. This led to an approach that blurred the boundaries between a legitimate referral to another level of care and a rejection of the patient as a person in need of care. Decisions were made over the older patient’s head, without considering the patient’s wishes and showed ignorance based on a perception that the patient was not receptive to information and participation. The risk of the patient not being heard meant a failing of the basis of the decision in the nurse’s assessment and that the patient should be cared for in a place other than the ED. In cases where the patient made himself heard, the risks of remaining in the ED were explained in a way that was not entirely accurate. For example, an unspoken consequence of delayed care was conveyed to steer the patient in a direction that the patient had not intended.

I also want them [the patients] to understand our business and it can be very difficult sometimes. So it can be frustrating when you do not understand each other I think. It can get a little hectic: ‘There is nothing you need to see a doctor for today, if nothing has worsened’. ‘Just today? But then you have to wait for seven-to-eight hours, do you want to?’

Understandings of legitimate care needs signified that acute nursing care was regarded as a narrow field of work where the older patient’s autonomy cannot be considered in the same way as in other care settings. The space provided for patient participation was limited and considered as a part of the planning of the care following the ED, and not the nursing care at the ED per se. Instead, to address patients’ expressed willingness for participation, information was provided with the purpose of getting the patients to understand the narrow objectives of the ED.

But he must understand that you do not come to the emergency room because you do not take care of your medication, and come here and get a ‘quick fix’ and then it’s okay. That’s not the point. Then I told him that ‘You have to take care of your medication, otherwise you will not be well’ or ‘You do not feel well not taking them’… So you can say that you made a decision for… Then I told him that ‘You must take care of your medication, because you do not take care of what you are actually obliged to do with your medication’.

## Discussion

The results describe nurses’ experiences of dealing with older patients’ autonomy as moving in a conflicting uphill struggle, indicating obscured thoughts on how to protect the patient’s autonomy in an ethically challenging context when prioritizing under stress. The nurses appeared to have an obscure understanding of autonomy and described their dealing with the older patient’s autonomy, even if they experienced several obstacles, both internal and external. Their fostering of older patients’ autonomy in the EDs was dependent on environmental circumstances and seemed to lack a developed structure for how to support and promote patient autonomy.

The present results indicate how an aggravating and stressful care environment with time pressures and lack of resources involve patients only to a small extent, and this thereby limits their autonomy, partly as a consequence of having limited space in which to listen to the patient’s will. Studies have indicated that older patients are especially vulnerable and their care needs are found to be de-prioritized in the ED environment,^5^ and, when excluded from care, this affects the patient’s integrity and dignity.^26^ Not respecting and listening to older patient’s needs and wishes is usually also contrary to the principle of autonomy and promoting good care.^19^ The ED nurses in the present study experienced stress and limited time as the most important obstacle to promoting the patient’s autonomy. Thus, relational aspects of autonomy became secondary because the nurses seemed to have a one-sided focus on medical, technical and routine measures, without, or with very limited, space for dialogue to hear the patient’s story. Such a paternalistic approach can be justified when it comes to making decisions for the patient’s best interests when his/her condition makes it impossible to obtain consent. The question is, however, how stress and lack of time should be balanced against an ethical demand to promote the older patient’s autonomy in cases where the patient’s ability to participate is fairly good. Based on relational autonomy,^8,16^ a commitment from nurses is required to support and promote patient autonomy in making judgements that are true to the patient’s own preferences. However, the prerequisite for such a commitment is largely based on establishing a responsible relationship between the nurse and the patient (and family members).

The present results reveal that older patients who are experienced as too demanding, based on the nurses’ notions of legitimate care needs to be met at the ED, are not involved in the nursing care. Consequently, no responsible relationship can be established, which in itself constitutes a threat to patient autonomy. Similar results have been found in a study where demanding patients were described as ‘bad patients’, which made the relationship difficult or uncomfortable. From the nurses’ perspective, this led to a strained relationship with lack of patient participation in care.^27^ In the present results, a strained relationship also relate to the nurses being exhausted. However, when patients struggle and make demands during their visit to the ED, studies show that it is usually a behaviour that should be interpreted as a desire to be involved,^28^ even if nurses associate such behaviour with being a ‘bad patient’.^27^ It may be that the ED nurses, due to their working conditions, do not have sufficient energy to be sensitive to patients’ unique expressions for participation. Although this may be an explanation for strained relationships, it is difficult to justify ignoring the fact that the patient is in an asymmetric relationship, as described by Delmar,^15^ where the nurse has power that should be handled responsibly.

The ED nurses also described conditions for a good care relationship and the patient’s opportunities for participation, even if, in their expert role, they had already decided what was best for patients, which is also called ‘protected paternalism’.^27^ This corresponds to the present results, as decisions were made without consent to provide lifesaving interventions, forcing the ED nurses to act based on what was believed to be in the patient’s best interests. The nurses described these decisions as temporary and necessary, as these patients cannot provide consent. Bremer et al.^29^ argue that, in acute situations, there may be uncertainty about the patient’s actual wishes if the patient is unable to participate, and if there are no written directives to follow. It then becomes a question of who is best suited to determine the best care decision for the patient. From a patient perspective, Rier^30^ argues that critically ill patients are unable to participate and negotiate, which makes informed consent impossible. Instead, acute and critical illness justifies paternalistic decisions about the patient, both on medical and on moral grounds.

The present findings show that older patients’ autonomy can be complex when there is no clear guidance, which requires that nurses take on a great deal of ethical responsibility along with a critical reflection on how the situation can be handled to be good and right. Edwards^31^ argues that doing good from a nursing perspective is to also take account of dimensions such as the relationship between the patient and family. According to Lindberg et al.,^32^ the prerequisites for the relationship differ when the patient is able to communicate but is very ill, and is able to hand over the choice and decision to the care staff. When the patient then recovers, their autonomy can be seen from the different aspect of co-determination, where the patient regains autonomy or, according to Holmberg et al.,^33^ experiences autonomy in surrendering to the healthcare professionals. In other studies,^30,34,35^ patients expressed their gratitude that the healthcare professionals acted paternalistically and thus saved their lives. Hence, there are multiple pathways to shared decision-making and the promotion of autonomy, which is further described by Sandman et al.^19^

The results show that paternalistic handling occurs through making decisions without consent, which entails a high risk of incorrect care actions, even if nurses judge that they are doing what is best for the older patient. Other studies have shown that nurses make decisions without consent in their expert role to achieve health-giving effects.^36,37^ However, although the nurse might be recognized as an expert from a professional point of view, the patient is the expert on his/her situation from a lifeworld perspective.^22,30^ When older patients are acutely or critically ill and have cognitive impairment, it has been found that nurses perceive themselves as being competent to make decisions without patient consent, even if the older patient should always be allowed and supported to participate in decision-making.^14^ This is unproblematic if nurses can also be open to the patient’s unique needs of being autonomous in different ways and to different degrees.^38^ Waiting for the patient’s actions and then reacting is a strategy described by healthcare professionals working in EDs. This further supports an overall question about who is responsible for the older patients’ autonomy: the professional, or the professional and the patient? In this regard, it has been shown that nurses prefer a submissive patient who assumes care without discussion.^39^ Thus, critical reflection and deeper understanding of the nuances and relational aspects of autonomy might be needed to support ED nurses in managing ethical values. However, in the present study, older patients’ autonomy was dealt with in an uncritical and non-reflective way, primarily when prioritizing under stress, which raises the question of whether ED nurses’ ethical competence needs to be supported in being ethically sensitive and ethically reflective to act in accordance with the patient’s preferences. Similar findings have shown how nurses may lack practical knowledge about how to act and in supporting the patient’s autonomy.^29^ Lack of time, ignorance and routine actions are risk factors for improper management of the older patient. Such behaviour risks unfair treatment and violations of integrity and dignity, especially as it has been shown that healthcare professionals find it particularly challenging to respect older patients.^26^

Furthermore, a lack of understanding of how to promote patient participation in an aggravating environment violates a fragile autonomy, leading to inadequacy in the nurses’ professional role. The messy environment in the EDs has been found to affect older patients’ cognitive capacity and erodes their cognitive ability and opportunities for participation.^40^ In this environment, nurses do not always ask patients about their involvement in care. One reason is that nurses may not believe that the patient is able to act as an autonomous person, reducing his/her participation. Conversely, nurses feel that they are the patients’ advocates,^41^ and thus must preserve the older patients’ ability to be autonomous. In this regard, previous studies identify nurses’ need for organizational^40^ and educational^29^ support and the development of strategies to support the patient and increase the patient’s autonomy.^29,40^

## Methodological considerations

The results of the study should be interpreted in light of some limitations. The interviews were conducted by two different interviewers who had limited experience of interviewing, which may have influenced the results. However, three pilot interviews were completed for training purposes before data collection began. These were then analysed by two senior experienced qualitative researchers. In a qualitative study, researchers should be aware of and should attempt to bridle their pre-understandings. Therefore, discussions between all researchers were conducted throughout the process to prevent such pre-understandings from introducing bias in the process.^19^ A constant dialogue took place between all researchers during the analysis, and the findings were discussed until consensus was reached. Trustworthiness was promoted by describing the research process carefully to make it possible for others to follow the process to transfer the results to other settings. The findings of this study might be transferable to other EDs where care follows a similar pattern, although the sample consists only of women. However, the relation between patients and healthcare staff may vary more across different countries and cultures. Therefore, similar studies in different cultures are warranted.

## Conclusion

Although ED nurses have an ambition to support the autonomy of older patients, the results indicate difficulties in relation to good intentions, but also feelings of powerlessness when nurses fall victim to circumstances they find difficult to influence. The nurses’ protection and promotion of older patients’ autonomy is dependent on the opportunity, ability and willingness to create a patient relationship where the patient’s voice and preferences are valued as important. Stressful work conditions and the lack of organizational strategies for promoting older patients’ autonomy in EDs contributes to maintaining unjustified paternalistic care and questioning who has a legitimate responsibility for participating in care decisions in the ED. Although nurses have ethical competence, strategies are needed to improve patient autonomy in EDs in order not to allow justified ‘protected paternalism’ to be applied in cases where the patient’s wishes should be given more space – based on the idea of ‘relational autonomy’.

## Highlights

• Promotion of patient autonomy is ethically challenging in emergency departments (ED)
• Emergency department nurses have obscure thoughts on how patient autonomy can be protected
• Emergency department nurses’ dealing with paternalism and patient autonomy are hampered by prioritization
• Emergency department nurses make decisions without consent in the patient’s best interests
• Emergency department nurses are being trapped by notions of legitimate care needs
